# Natural history of subclinical hypothyroidism in children and adolescents

**DOI:** 10.1186/1756-6614-6-S2-A41

**Published:** 2013-04-05

**Authors:** Ewa Małecka-Tendera

**Affiliations:** 1 Department of Pediatrics, Pediatric Endocrinology and Diabetes, Medical University of Silesia, Katowice, Poland

## 

Diagnosis of subclinical hypothyroidism (SH) is based on interpretation of biochemical tests in the absence of the evident clinical symptoms. Mildly elevated TSH with normal fT_4_ are common in adults and the prevalence of this finding is reported to be 1-10% of general population, being higher in the elderly. In pediatric population its prevalence is lower than 2%. Moreover in about 60% of subjects the natural course of SH is a reversal of the elevated TSH to normal values. Only 3% of them progress to overt hypothyroidism with TSH values above 10 mUI/l. The risk of progression is higher in patients with elevated anti-thyroid antibodies and higher degree of hypoechogenicity at thyroid ultrasound. Increased prevalence of SH is described in obese and overweight subjects, children with Down’s syndrome, with diabetes type 1 and in girls with Turner’s syndrome. Studies regarding the natural history of SH and its consequences in children are scarce and their conclusions are controversial. Meta-analysis of 39 potentially relevant articles showed that SH in children seems to be a remitting process with a low risk of progression toward overt hypothyroidism regardless of the L-T _4_ treatment. There was also no clear evidence of the beneficial effect of L-T_4_ treatment on psychological and physical development. Replacement therapy did not seem to be justified in children with SH and TSH values between 5 -10 mUI/l, no goiter and negative anti-thyroid antibodies. Therefore decision regarding the treatment of the young patient with elevated TSH but normal fT_4_ value continues to be controversial.

